# Two coordination compounds of SnCl_2_ with 4-methyl­pyridine *N*-oxide

**DOI:** 10.1107/S2056989021000025

**Published:** 2021-01-08

**Authors:** Felix Henkel, Hans Reuter

**Affiliations:** a Institute of Chemistry of New Materials, University of Osnabrück, Barbarastr. 7, 49069 Osnabrück, Germany

**Keywords:** crystal structure, SnCl_2_ coordination compounds, 4-methyl­pyridine *N*-oxide, bond-valence calculations, coordination geometry

## Abstract

In the solid-state structures of *catena*-poly[[di­chlorido­tin(II)]-μ_2_-(4-methyl­pyridine *N*-oxide)-κ^2^ *O*:*O*], [SnCl_2_(C_6_H_7_NO)]_*n*_, **1**, and di­chlorido­bis­(4-methyl­pyridine *N*-oxide-κ*O*)tin(II), [SnCl_2_(C_6_H_7_NO)_2_], **2**, the bivalent tin atoms reveal a seesaw coordination with both chlorine atoms in equatorial and the Lewis base mol­ecules in axial positions. While the Sn—Cl distances are almost identical, the Sn—O distances vary significantly as a result of the different bonding modes (μ_2_ for **1**, μ_1_ for **2**) of the 4-methyl­pyridin-*N*-oxide mol­ecules, giving rise to a one-dimensional coordination polymer for the 1:1 adduct, **1**, and a mol­ecular structure for the 1:2 adduct, **2**. The different coordination modes also influence the bonding parameters within the almost planar ligand mol­ecules, mostly expressed in N—O-bond lengthening and endocyclic bond-angle widening at the nitro­gen atoms. Additional supra­molecular features are found in the crystal structure of **2** as two adjacent mol­ecules form dimers *via* additional, weak O⋯Sn inter­actions.

## Abstract 2

Single crystals of SnCl_2_·MePyNO, **1**, and SnCl_2_·2MePyNO, **2**, have been synthesized and structurally characterized by X-ray diffraction to investigate the structural changes of MePyNO as a result of its different coordination modes and the variation of the coordination geometry at the tin(II) atoms.

## Chemical context   

Tin(II) halides, SnHal_2_, are nominally electron-deficient compounds and therefore strong Lewis acids. Corresponding Lewis acid/Lewis base adducts, however, have been structurally characterized in only small numbers so far. Examples are known with Lewis base mol­ecules bearing nitro­gen [SnCl_2_·^*t*^BuNH_2_ (Veith *et al.*, 1988 ▸)], phospho­rus [SnCl_2_·Ph_3_P (Lukic *et al.*, 2019 ▸); SnHal_2_·Et_3_P (Arp *et al.*, 2013 ▸)], or sulfur [SnCl_2_·thio­urea (Harrison *et al.*, 1983 ▸)] atoms as possible donor atoms, but the most prominent ones are those with oxygen atoms. Tri­phenyl­phosphine oxide (TPPO), di­methyl­sulfoxide (DMSO) and *N*,*N*-di­methyl­formide (DMF) are representative examples for such O-bearing Lewis base mol­ecules. Typically, the tin(II) dihalides form 1:1 adducts [*e.g*. SnHal_2_·DMF with Hal = Cl, Br, I, and SnI_2_·DMSO (Ozaki *et al.*, 2017 ▸)] where the tin(II) atoms in these complexes reach an electron octet by taking up the two donor electrons of the Lewis base mol­ecule. In the case of 1:2 adducts [*e.g.* SnF_2_·2DMSO (Gurnani *et al.*, 2013 ▸); SnCl_2_·2TPPO (Selvaraju *et al.*, 1998 ▸); SnCl_2_·2DMSO (Barbul *et al.*, 2011 ▸); SnBr_2_·2DMSO, SnBr_2_·2THF, SnBr_2_·2acetone (Schrenk *et al.*, 2009 ▸)] the tin(II) atoms exceed the electron octet as a result of the two additional donor electrons. Both 1:1 and 1:2 compositions of one and the same tin(II) halide with one and the same Lewis base mol­ecule have been previously reported only for SnI_2_ with DMSO (Ozaki *et al.*, 2017 ▸).

Pyridin-*N*-oxide, PyNO, and its derivatives such as 4-methyl­pyridin-*N*-oxide, MePyNO, are excellent Lewis bases, which act as electron-pair donors *via* their exocyclic single-bonded oxygen atom in numerous inorganic and organometallic compounds of transition metals [*i.e.* CdHal_2_·PyNO with Hal = Cl (Beyeh & Puttreddy, 2015 ▸), Hal = I (Sawitzki & von Schnering, 1974 ▸), CuCl_2_·2MePyNO (Johnson & Watson, 1971 ▸), Ni(BF_4_)_2_·6PyNO (Ingen Schenau *et al.*, 1974 ▸), Au(CF_3_)_3_·PyNO (Pérez-Bitrián *et al.*, 2017 ▸), MoO(O_2_)_2_·2MePyNO (Griffith *et al.*, 1994 ▸)] as well as of *p*-block metals [*i.e*. TlBr_3_·PyNO (Bermejo *et al.*, 1991 ▸); TlBr_3_·2PyNO (Hiller *et al.*, 1988 ▸); TlBrI_2_·MePyNO (Hiller *et al.*, 1988 ▸); SnI_4_·2PyNO (Wlaźlak *et al.*, 2016 ▸), Me_2_SnCl_2_·2PyNO (Blom *et al.*, 1969 ▸), Ph_3_SnCl·PyNO (Kumar *et al.*, 2020 ▸). With the exception of SbF_3_·PyNO (Benjamin *et al.*, 2012 ▸) and BiI_3_·PyNO (Wlaźlak *et al.*, 2020 ▸), no complexes of low-valent post-transition-metal elements have been crystallographically determined so far.
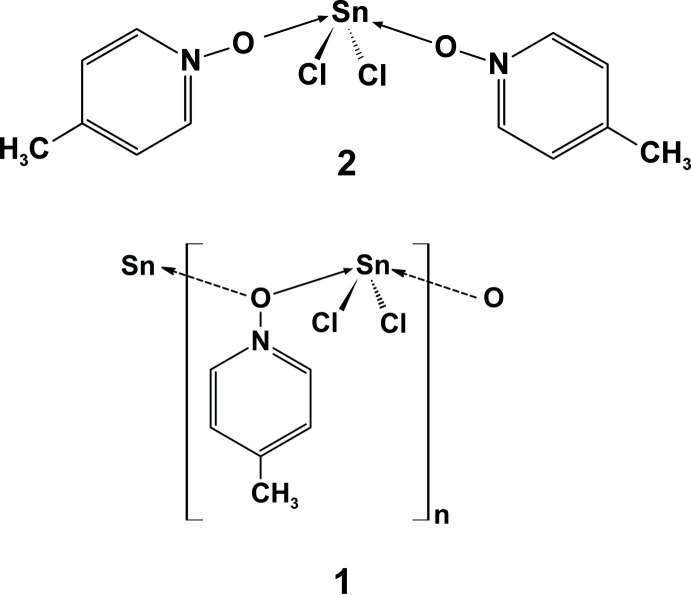


Here we report the crystal structures of two complexes of MePyNO with tin in oxidation state +II having different compositions, *viz*. SnCl_2_·MePyNO, **1**, and SnCl_2_·2MePyNO, **2**. Both compounds were obtained simultaneously in the same micro-scale experiment from SnCl_2_ and MePyNO in excess using *N,N*-di­methyl­formamide as solvent. As reactions were performed on reaction plates we were able to inspect the progress of the reaction by microscopy, which allowed us to observe the inter­mediate compound formation as well as to study the crystal growth. No scaling-up experiments were performed but **1** has previously been mentioned in the literature with respect to its elemental analysis, X-ray-powder diffraction and IR data (Kauffman *et al.*, 1977 ▸), giving hints of a low symmetric crystal system and coordination number of three for tin. Mössbauer investigations have been performed by Ichiba *et al.* (1978 ▸).

## Structural commentary   

**Figure 1:**
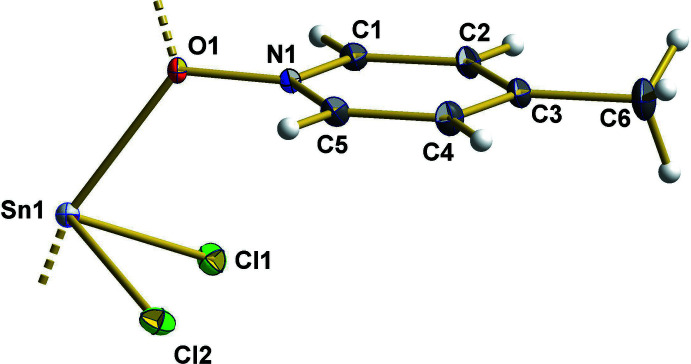
The asymmetric unit of SnCl_2_·MePyNO, **1**, with the atom-numbering scheme; with the exception of the hydrogen atoms (which are shown as spheres with arbitrary radius) all atoms are drawn with displacement ellipsoids at the 40% probability level; longer Sn—O bonds expanding the coordination sphere of the tin(II) atom from three, trigonal–pyramidal, to four, seesaw, are drawn as dashed sticks.

**Figure 2:**
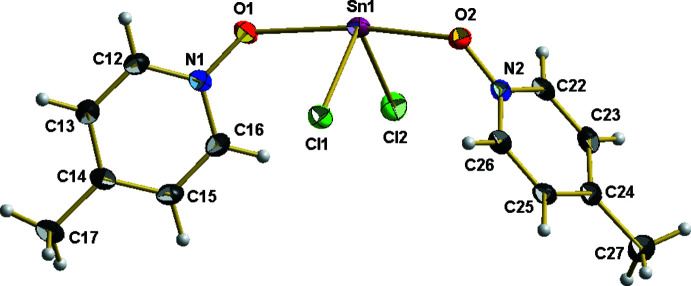
The asymmetric unit of SnCl_2_·2MePyNO, **2**, with the atom-numbering scheme; with exception of the hydrogen atoms (which are shown as spheres with arbitrary radius) all atoms are drawn with displacement ellipsoids at the 40% probability level.

Compound **1** crystallizes in the monoclinic space group *P*2_1_/c, and **2** in the ortho­rhom­bic space group *Pbcn*, each with one formula unit in the asymmetric unit and all atoms in general positions. In both compounds, the bivalent tin atoms adopt a seesaw coordination, which results from a *μ*_2_-coordination mode of the MePyNO-mol­ecule in **1**, giving rise to a one-dimensional coordination polymer along the *c* axis (Fig. 1 ▸) while there are two crystallographically different MePyNO mol­ecules in **2**, resulting in a mol­ecular structure (Fig. 2 ▸).

Distortion of the pyridine *N*-oxide ring system as a result of its coordination is established through the C—C [mean values: *d*(C_*ortho*_—C_*meta*_) = 1.376 (1) Å, *d*(C_*meta*_—C_*para*_) = 1.394 (3) Å] and the N—C bond lengths [mean value: *d*(N—C) = 1.347 (3) Å], and through the endocyclic bond angles at the different carbon atoms [mean values: C_*ortho*_ = 120.0 (3)°, C_*meta*_ = 120.8 (2)°, C_*para*_ = 117.1 (2)°] of the almost planar ligand. While these structural parameters are almost identical in both compounds, the N—O bond lengths differ significantly in **1** [1.363 (2) Å] and **2** [1.333 (3)/1.340 (3) Å] as do the endocyclic bond angles [121.9 (1)°, **1**; 120.9 (1)°, **2**] at the N atom. Both effects result from the different (*μ*_2_, *μ*_1_) coordination modes of the ligands, which also affect the Sn—O bond lengths that are strongly unsymmetrical [2.280 (1) to 2.733 (2) Å, *μ*_2_] in **1**, and less unsymmetrical [2.308 (2) to 2.423 (2) Å, *μ*_1_] in **2**.

Irrespective of the controversial discussion on the hybrid­ization ability of atomic orbitals in the case of the heavier *p*-block elements (Kutzelnigg, 1984 ▸), the formation of four-electron three-center bonds (Rundle, 1963 ▸), and on the functionality of the so-called 5*s* lone electron pair (Dénes *et al.*, 2013 ▸) in hypervalent (Musher, 1969 ▸) tin(II) compounds, the fourfold coordination sphere around the tin(II) atoms of **1** and **2** can be expressed very well in terms of the VSEPR concept (Gillespie & Hargittai, 1991 ▸): its seesaw (*ss*) coordination results from two equatorially bonded chlorine atoms and two more electronegative and therefore axially located oxygen atoms of the Lewis base, MePyNO.

Differences in Sn—Cl distances are very small [2.4850 (4) and 2.4905 (4) Å, **1**; 2.4939 (6) and 2.5068 (6) Å, **2**, mean value: 2.494 (9) Å] as are the bond angles [95.73 (1)°, **1**; 94.59 (2)°, **2**] between them. Somewhat shorter Sn—Cl distances are found in the six crystallographically independent mol­ecules of SnCl_2_·DMF [*d*(Sn—Cl)_mean_ = 2.458 (21) Å, 〈(Cl—Sn—Cl) = 92.89 (7)–89.09 (7)°] with a predominant trigonal–pyramidal coordination at tin, while the values in SnCl_2_·2DMSO [*d*(Sn—Cl)_mean_ = 2.483 (8) Å, 〈(Cl—Sn—Cl) = 93.86 (7)°] with a symmetrical seesaw coordination are in between.

Axes of the seesaws are bent [161.40 (6)°, **1**; 169.66 (6)°, **2**] towards the chlorine atoms properly due to electronic repulsion of the axial bonds through the 5*s* free-electron pairs. The corresponding Sn—O bonds are strongly different in both compounds, but differences are more expressed in **1** [2.280 (1) to 2.733 (1) Å, *μ*_2_-O] than in **2** [2.308 (2) to 2.430 (2) Å, *μ*_1_-O]. Because of the great [0.453 Å] difference between the two Sn—O bonds in **1**, one may suggest a threefold trigonal–pyramidal (*tpy*) tin(II) coordination instead of a fourfold seesaw (*ss*) coordination but valence-bond-sum calculations [parameters used: *r*_o_(Sn^II^—O) = 1.984 Å, *r*_o_(Sn^II^—Cl) = 2.335 Å, *b* = 0.37; Brese & O’Keefe (1991 ▸)] on the *tpy* coord­ination result in a bond-valence sum of 1.78 v.u. while the longer Sn—O bond in the *ss*-coordination contributes 0.13 v.u. to the bond-valence sum (1.91 v.u.). Similar calculations for **2** result in a bond-valence sum of 2.00 v.u., fully consistent with the tin oxidation state of +II.

## Supra­molecular features   

**Figure 3:**
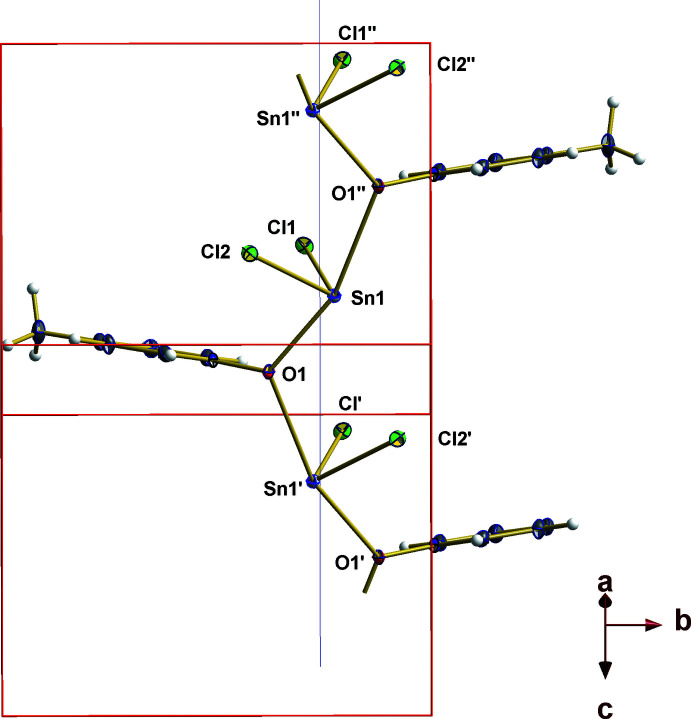
Ball-and-stick model of the one-dimensional coordination polymer of **1** viewed parallel to the glide plane (blue line); symmetry codes used to generate equivalent atoms: (′) *x*, 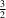 − *y*, 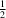 + *z*; (’’) *x*, 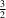 − *y*, −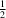 + *z*.

A common feature of many tin(II) compounds is the non-spherical ligand distribution around the divalent tin atom for which the term ‘hemidirected’ has been introduced (Shimoni-Livny *et al.*, 1998 ▸). The resulting void in the hemidirected coordination sphere often gives rise to additional more or less weak inter­molecular (and intra­molecular if appropriate Lewis base donor atoms are sterically available) inter­actions with inter­esting supra­molecular features. In case of **1**, the formation of a one-dimensional coordination polymer *via* the *μ*_2_-O-atom of the MePyNO mol­ecule can be inter­preted in terms of such supra­molecular inter­actions: in this particular case, the hemidirected coordination sphere of a mol­ecular, trigonal–pyramidal SnCl_2_·MePyNO complex is partially filled through the oxygen atom of a MePyNO mol­ecule of a neighboring building unit. The resulting coordination polymer forms a zigzag chain as all atoms are situated off the crystallographic glide plane at *x*, 1/4, *z* (Fig. 3 ▸). Between the zigzag chains no further Lewis base/Lewis acid inter­actions below 3.5 Å are observed, but within the chains a very weak [3.460 (1) Å] attractive inter­action is found between Cl2 and Sn1 of two neighboring building units (Fig. 3 ▸).

**Figure 4:**
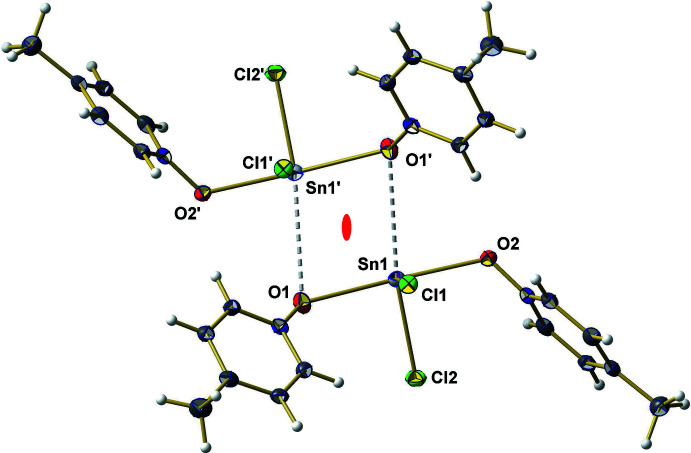
Ball-and-stick model of the dimeric aggregates found in the crystal structure of **2** looking down the crystallographic twofold rotation axis marked in red; additional Sn—O distances are indicated by dashed sticks in gray [symmetry codes used to generate equivalent atoms marked ′: 1 − *x*, *y*, 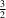 − *z*.]

**Figure 5:**
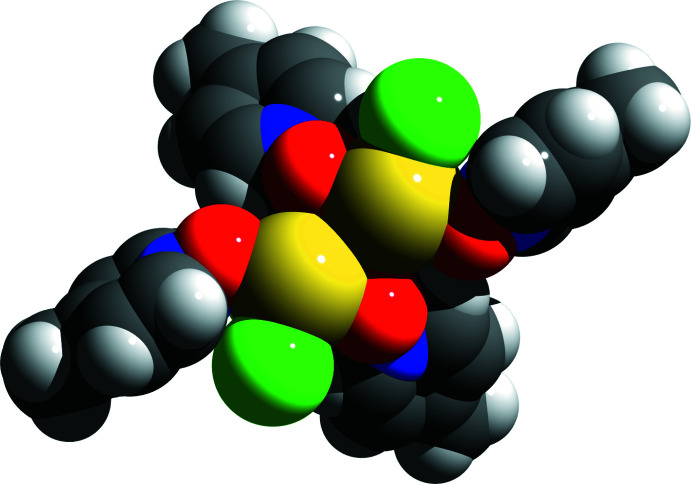
Space-filling model of the dimeric aggregates found in the crystal structure of **2** looking down into the voids on the backside of the tin atoms; color code used: Cl = green, O = red, N = blue, C = black, H = gray, Sn = yellow.

In case of **2** the tin atom of the SnCl_2_·2MePyNO mol­ecules shows a similar hemidirected coordination sphere. In the solid state, neighboring mol­ecules form dimers *via* attractive but very weak [3.225 (2) Å] Sn—O inter­actions. Both mol­ecules of these dimers are related to each other *via* a crystallographic twofold rotation axis (Fig. 4 ▸). Even if the coordination sphere of each tin atom remains unsymmetrical in these dimeric aggregates (Fig. 5 ▸), no further inter­molecular inter­actions could be observed below 3.5 Å.

## Synthesis and crystallization   

Both complexes are formed side by side on a reaction plate in the same micro-scale experiment when small amounts (about 100 mg) of SnCl_2_ (Sigma–Aldrich) and an excess of 4-MePyNO (Alfa Aesar) are overlaid with a few drops of *N,N*-di­methyl­formamide (Fluka) as solvent. Compound **1** forms colorless, elongated plates, while **2** crystallizes in the form of small, colorless prisms.

## Refinement   

Crystal data, data collection and structure refinement details of **1** and **2** are summarized in Table 1 ▸. All H atoms were clearly identified in difference-Fourier syntheses but were refined with idealized positions and allowed to ride on their parent carbon atoms with 0.98 Å (–CH_3_), and 0.95 Å (–CH_arom_) and with common isotropic temperature factors for all hydrogen atoms of the aromatic rings and methyl groups.

## Supplementary Material

Crystal structure: contains datablock(s) 1, 2. DOI: 10.1107/S2056989021000025/wm5593sup1.cif

Structure factors: contains datablock(s) 1. DOI: 10.1107/S2056989021000025/wm55931sup2.hkl

Structure factors: contains datablock(s) 2. DOI: 10.1107/S2056989021000025/wm55932sup3.hkl

CCDC references: 2053460, 2053459

Additional supporting information:  crystallographic information; 3D view; checkCIF report

## Figures and Tables

**Table 1:** Experimental details

	**1**	**2**
Crystal data		
Chemical formula	[SnCl_2_(C_6_H_7_NO)]	[SnCl_2_(C_6_H_7_NO)_2_]
*M*_r_	298.72	407.84
Crystal system, space group	Monoclinic, *P*2_1_/*c*	Orthorhombic, *P* *b* *c* *n*
Temperature (K)	100	100
*a*, *b*, *c* (Å)	11.7934 (4), 9.4895 (3), 8.6170 (3)	19.9848 (8), 10.3723 (3), 14.4644 (5)
α, β, γ (°)	90, 106.455 (2), 90	90, 90, 90
*V* (Å^3^)	924.86 (5)	2998.30 (18)
*Z*	4	8
Radiation type	Mo *K*α	Mo *K*α
μ (mm^−1^)	3.28	2.06
Crystal size (mm)	0.49 × 0.17 × 0.06	0.47 × 0.11 × 0.07

Data collection		
Diffractometer	Bruker APEXII CCD	Bruker APEXII CCD
Absorption correction	Multi-scan (*SADABS*; Krause *et al.*, 2015 ▸)	Multi-scan (*SADABS*; Krause *et al.*, 2015 ▸)
*T*_min_, *T*_max_	0.298, 0.817	0.442, 0.866
No. of measured, independent and observed [*I* > 2σ(*I*)] reflections	86401, 2232, 2132	136234, 3626, 3086
*R*_int_	0.039	0.090
(sin θ/λ)_max_ (Å^−1^)	0.661	0.660

Refinement		
*R*[*F* ^2^ > 2σ(*F* ^2^)], *wR*(*F* ^2^), *S*	0.013, 0.032, 1.12	0.025, 0.064, 1.10
No. of reflections	2232	3626
No. of parameters	103	178
H-atom treatment	H-atom parameters constrained	H-atom parameters constrained
Δρ_max_, Δρ_min_ (e Å^−3^)	0.41, −0.49	0.81, −0.33

Computer programs: *APEX2* and *SAINT* (Bruker, 2009 ▸), *SHELXS97* (Sheldrick, 2008 ▸), *SHELXL2014/7* (Sheldrick, 2015 ▸), *DIAMOND* (Brandenburg, 2006 ▸), *Mercury* (Macrae *et al.*, 2020 ▸) and *publCIF* (Westrip, 2010 ▸).

## supplementary crystallographic information

### catena-Poly[[dichloridotin(II)]-µ2-(4-methylpyridine N-oxide)-κ2O:O] (1) Crystal data

**Table:**

[SnCl_2_(C_6_H_7_NO)]	*F*(000) = 568
*M**_r_* = 298.72	*D*_x_ = 2.145 Mg m^−^^3^
Monoclinic, *P*2_1_/*c*	Mo *K*α radiation, λ = 0.71073 Å
*a* = 11.7934 (4) Å	Cell parameters from 9441 reflections
*b* = 9.4895 (3) Å	θ = 2.8–28.3°
*c* = 8.6170 (3) Å	µ = 3.28 mm^−^^1^
β = 106.455 (2)°	*T* = 100 K
*V* = 924.86 (5) Å^3^	Plate, colourless
*Z* = 4	0.49 × 0.17 × 0.06 mm

### catena-Poly[[dichloridotin(II)]-µ2-(4-methylpyridine N-oxide)-κ2O:O] (1) Data collection

**Table:**

Bruker APEXII CCD diffractometer	2132 reflections with *I* > 2σ(*I*)
φ and ω scans	*R*_int_ = 0.039
Absorption correction: multi-scan (SADABS; Krause *et al.*, 2015)	θ_max_ = 28.0°, θ_min_ = 3.3°
*T*_min_ = 0.298, *T*_max_ = 0.817	*h* = −15→15
86401 measured reflections	*k* = −12→12
2232 independent reflections	*l* = −11→11

### catena-Poly[[dichloridotin(II)]-µ2-(4-methylpyridine N-oxide)-κ2O:O] (1) Refinement

**Table:**

Refinement on *F*^2^	0 restraints
Least-squares matrix: full	Hydrogen site location: inferred from neighbouring sites
*R*[*F*^2^ > 2σ(*F*^2^)] = 0.013	H-atom parameters constrained
*wR*(*F*^2^) = 0.032	*w* = 1/[σ^2^(*F*_o_^2^) + (0.0134*P*)^2^ + 0.6437*P*] where *P* = (*F*_o_^2^ + 2*F*_c_^2^)/3
*S* = 1.12	(Δ/σ)_max_ = 0.003
2232 reflections	Δρ_max_ = 0.41 e Å^−^^3^
103 parameters	Δρ_min_ = −0.49 e Å^−^^3^

### catena-Poly[[dichloridotin(II)]-µ2-(4-methylpyridine N-oxide)-κ2O:O] (1) Special details

**Table:**

Geometry. All esds (except the esd in the dihedral angle between two l.s. planes) are estimated using the full covariance matrix. The cell esds are taken into account individually in the estimation of esds in distances, angles and torsion angles; correlations between esds in cell parameters are only used when they are defined by crystal symmetry. An approximate (isotropic) treatment of cell esds is used for estimating esds involving l.s. planes.

### catena-Poly[[dichloridotin(II)]-µ2-(4-methylpyridine N-oxide)-κ2O:O] (1) Fractional atomic coordinates and isotropic or equivalent isotropic displacement parameters (Å^2^)

**Table:**

	*x*	*y*	*z*	*U*_iso_*/*U*_eq_	
Sn1	0.65817 (2)	0.77494 (2)	0.40278 (2)	0.01249 (4)	
Cl1	0.86766 (3)	0.70601 (4)	0.43743 (5)	0.01738 (8)	
Cl2	0.57006 (3)	0.57820 (4)	0.21746 (4)	0.01829 (8)	
O1	0.65673 (9)	0.62193 (11)	0.60614 (13)	0.0130 (2)	
N1	0.70802 (11)	0.49228 (13)	0.61676 (15)	0.0114 (2)	
C2	0.82254 (13)	0.47923 (17)	0.70246 (18)	0.0142 (3)	
H2	0.8666	0.5598	0.7501	0.021 (2)*	
C3	0.87519 (14)	0.34877 (17)	0.72050 (19)	0.0157 (3)	
H3	0.9558	0.3397	0.7812	0.021 (2)*	
C4	0.81191 (14)	0.22964 (16)	0.65083 (19)	0.0146 (3)	
C5	0.69385 (14)	0.24895 (17)	0.5615 (2)	0.0166 (3)	
H5	0.6482	0.1704	0.5111	0.021 (2)*	
C6	0.64331 (13)	0.38104 (16)	0.54603 (18)	0.0143 (3)	
H6	0.5630	0.3934	0.4856	0.021 (2)*	
C7	0.86871 (15)	0.08689 (17)	0.6700 (2)	0.0215 (3)	
H71	0.9108	0.0742	0.5880	0.051 (4)*	
H72	0.8077	0.0141	0.6566	0.051 (4)*	
H73	0.9247	0.0790	0.7781	0.051 (4)*	

### catena-Poly[[dichloridotin(II)]-µ2-(4-methylpyridine N-oxide)-κ2O:O] (1) Atomic displacement parameters (Å^2^)

**Table:**

	*U*^11^	*U*^22^	*U*^33^	*U*^12^	*U*^13^	*U*^23^
Sn1	0.01487 (6)	0.01142 (6)	0.01145 (6)	0.00176 (4)	0.00415 (4)	0.00048 (4)
Cl1	0.01430 (17)	0.01858 (18)	0.01902 (18)	0.00012 (14)	0.00435 (14)	0.00240 (14)
Cl2	0.02132 (18)	0.01852 (18)	0.01377 (17)	−0.00592 (15)	0.00295 (14)	−0.00073 (14)
O1	0.0161 (5)	0.0100 (5)	0.0138 (5)	0.0050 (4)	0.0055 (4)	0.0021 (4)
N1	0.0125 (6)	0.0099 (6)	0.0120 (6)	0.0021 (5)	0.0036 (5)	0.0018 (5)
C2	0.0134 (7)	0.0136 (7)	0.0142 (7)	−0.0013 (6)	0.0018 (6)	−0.0007 (6)
C3	0.0123 (7)	0.0157 (7)	0.0170 (7)	0.0016 (6)	0.0008 (6)	0.0017 (6)
C4	0.0157 (7)	0.0113 (7)	0.0163 (7)	0.0009 (6)	0.0038 (6)	0.0012 (6)
C5	0.0145 (7)	0.0133 (7)	0.0197 (8)	−0.0025 (6)	0.0011 (6)	−0.0009 (6)
C6	0.0122 (7)	0.0146 (7)	0.0152 (7)	−0.0018 (6)	0.0023 (6)	0.0008 (6)
C7	0.0194 (8)	0.0121 (7)	0.0300 (9)	0.0024 (6)	0.0022 (7)	0.0006 (7)

### catena-Poly[[dichloridotin(II)]-µ2-(4-methylpyridine N-oxide)-κ2O:O] (1) Geometric parameters (Å, º)

**Table:**

Sn1—O1	2.2795 (10)	C3—H3	0.9500
Sn1—Cl2	2.4850 (4)	C4—C5	1.399 (2)
Sn1—Cl1	2.4905 (4)	C4—C7	1.499 (2)
O1—N1	1.3626 (16)	C5—C6	1.378 (2)
N1—C6	1.343 (2)	C5—H5	0.9500
N1—C2	1.3490 (19)	C6—H6	0.9500
C2—C3	1.374 (2)	C7—H71	0.9800
C2—H2	0.9500	C7—H72	0.9800
C3—C4	1.393 (2)	C7—H73	0.9800
			
O1—Sn1—Cl2	85.56 (3)	C3—C4—C7	121.16 (14)
O1—Sn1—Cl1	87.92 (3)	C5—C4—C7	121.53 (14)
Cl2—Sn1—Cl1	95.726 (14)	C6—C5—C4	120.54 (15)
N1—O1—Sn1	121.80 (8)	C6—C5—H5	119.7
C6—N1—C2	121.88 (13)	C4—C5—H5	119.7
C6—N1—O1	119.66 (12)	N1—C6—C5	119.76 (14)
C2—N1—O1	118.44 (12)	N1—C6—H6	120.1
N1—C2—C3	119.67 (14)	C5—C6—H6	120.1
N1—C2—H2	120.2	C4—C7—H71	109.5
C3—C2—H2	120.2	C4—C7—H72	109.5
C2—C3—C4	120.83 (14)	H71—C7—H72	109.5
C2—C3—H3	119.6	C4—C7—H73	109.5
C4—C3—H3	119.6	H71—C7—H73	109.5
C3—C4—C5	117.31 (14)	H72—C7—H73	109.5

### Dichloridobis(4-methylpyridine N-oxide-κO)tin(II) (2) Crystal data

**Table:**

[SnCl_2_(C_6_H_7_NO)_2_]	*D*_x_ = 1.807 Mg m^−^^3^
*M**_r_* = 407.84	Mo *K*α radiation, λ = 0.71073 Å
Orthorhombic, *P**b**c**n*	Cell parameters from 9991 reflections
*a* = 19.9848 (8) Å	θ = 2.6–27.2°
*b* = 10.3723 (3) Å	µ = 2.06 mm^−^^1^
*c* = 14.4644 (5) Å	*T* = 100 K
*V* = 2998.30 (18) Å^3^	Rod, colourless
*Z* = 8	0.47 × 0.11 × 0.07 mm
*F*(000) = 1600	

### Dichloridobis(4-methylpyridine N-oxide-κO)tin(II) (2) Data collection

**Table:**

Bruker APEXII CCD diffractometer	3086 reflections with *I* > 2σ(*I*)
φ and ω scans	*R*_int_ = 0.090
Absorption correction: multi-scan (SADABS; Krause *et al.*, 2015)	θ_max_ = 28.0°, θ_min_ = 2.6°
*T*_min_ = 0.442, *T*_max_ = 0.866	*h* = −26→26
136234 measured reflections	*k* = −13→13
3626 independent reflections	*l* = −19→19

### Dichloridobis(4-methylpyridine N-oxide-κO)tin(II) (2) Refinement

**Table:**

Refinement on *F*^2^	0 restraints
Least-squares matrix: full	Hydrogen site location: inferred from neighbouring sites
*R*[*F*^2^ > 2σ(*F*^2^)] = 0.025	H-atom parameters constrained
*wR*(*F*^2^) = 0.064	*w* = 1/[σ^2^(*F*_o_^2^) + (0.0279*P*)^2^ + 2.7839*P*] where *P* = (*F*_o_^2^ + 2*F*_c_^2^)/3
*S* = 1.10	(Δ/σ)_max_ = 0.001
3626 reflections	Δρ_max_ = 0.81 e Å^−^^3^
178 parameters	Δρ_min_ = −0.33 e Å^−^^3^

### Dichloridobis(4-methylpyridine N-oxide-κO)tin(II) (2) Special details

**Table:**

Geometry. All esds (except the esd in the dihedral angle between two l.s. planes) are estimated using the full covariance matrix. The cell esds are taken into account individually in the estimation of esds in distances, angles and torsion angles; correlations between esds in cell parameters are only used when they are defined by crystal symmetry. An approximate (isotropic) treatment of cell esds is used for estimating esds involving l.s. planes.

### Dichloridobis(4-methylpyridine N-oxide-κO)tin(II) (2) Fractional atomic coordinates and isotropic or equivalent isotropic displacement parameters (Å^2^)

**Table:**

	*x*	*y*	*z*	*U*_iso_*/*U*_eq_	
Sn1	0.55158 (2)	0.16754 (2)	0.64760 (2)	0.02085 (6)	
Cl1	0.55438 (3)	0.40624 (6)	0.62730 (4)	0.02477 (13)	
Cl2	0.66633 (3)	0.13226 (7)	0.58237 (5)	0.03087 (14)	
O1	0.60299 (9)	0.21147 (17)	0.79652 (12)	0.0288 (4)	
N1	0.63657 (10)	0.31475 (19)	0.82579 (14)	0.0218 (4)	
C12	0.62539 (12)	0.3580 (2)	0.91192 (17)	0.0229 (5)	
H12	0.5917	0.3191	0.9489	0.029 (4)*	
C13	0.66263 (11)	0.4585 (2)	0.94668 (17)	0.0231 (5)	
H13	0.6548	0.4874	1.0081	0.029 (4)*	
C14	0.71145 (12)	0.5186 (2)	0.89344 (17)	0.0233 (5)	
C15	0.71995 (11)	0.4734 (2)	0.80348 (17)	0.0242 (5)	
H15	0.7518	0.5135	0.7641	0.029 (4)*	
C16	0.68266 (12)	0.3714 (2)	0.77119 (17)	0.0243 (5)	
H16	0.6894	0.3407	0.7100	0.029 (4)*	
C17	0.75292 (14)	0.6270 (3)	0.9311 (2)	0.0335 (6)	
H17A	0.7345	0.7095	0.9100	0.068 (7)*	
H17B	0.7991	0.6183	0.9092	0.068 (7)*	
H17C	0.7523	0.6241	0.9989	0.068 (7)*	
O2	0.50452 (9)	0.16530 (16)	0.50191 (12)	0.0236 (4)	
N2	0.53989 (9)	0.18673 (19)	0.42470 (13)	0.0193 (4)	
C22	0.57330 (12)	0.0882 (2)	0.38493 (17)	0.0218 (5)	
H22	0.5738	0.0058	0.4137	0.023 (3)*	
C23	0.60658 (11)	0.1070 (2)	0.30287 (17)	0.0236 (5)	
H23	0.6299	0.0371	0.2753	0.023 (3)*	
C24	0.60656 (12)	0.2270 (2)	0.25955 (17)	0.0235 (5)	
C25	0.57304 (13)	0.3268 (2)	0.30435 (17)	0.0230 (5)	
H25	0.5728	0.4106	0.2778	0.023 (3)*	
C26	0.54027 (12)	0.3055 (2)	0.38668 (18)	0.0214 (5)	
H26	0.5179	0.3745	0.4168	0.023 (3)*	
C27	0.64072 (15)	0.2477 (3)	0.16818 (19)	0.0350 (6)	
H27A	0.6890	0.2567	0.1780	0.097 (10)*	
H27B	0.6233	0.3261	0.1392	0.097 (10)*	
H27C	0.6322	0.1736	0.1278	0.097 (10)*	

### Dichloridobis(4-methylpyridine N-oxide-κO)tin(II) (2) Atomic displacement parameters (Å^2^)

**Table:**

	*U*^11^	*U*^22^	*U*^33^	*U*^12^	*U*^13^	*U*^23^
Sn1	0.02042 (9)	0.01901 (10)	0.02311 (9)	−0.00041 (6)	0.00035 (6)	0.00205 (6)
Cl1	0.0277 (3)	0.0191 (3)	0.0275 (3)	0.0016 (2)	0.0005 (2)	0.0005 (2)
Cl2	0.0220 (3)	0.0358 (3)	0.0348 (3)	0.0041 (2)	0.0026 (2)	−0.0067 (3)
O1	0.0351 (10)	0.0244 (9)	0.0270 (9)	−0.0098 (8)	−0.0062 (8)	0.0017 (7)
N1	0.0213 (10)	0.0195 (10)	0.0247 (10)	−0.0023 (8)	−0.0028 (8)	0.0013 (8)
C12	0.0193 (11)	0.0250 (12)	0.0243 (12)	0.0016 (9)	0.0014 (9)	0.0039 (9)
C13	0.0218 (11)	0.0237 (12)	0.0240 (11)	0.0028 (9)	0.0002 (9)	0.0005 (9)
C14	0.0208 (11)	0.0191 (12)	0.0298 (12)	0.0008 (9)	−0.0008 (10)	0.0018 (10)
C15	0.0177 (10)	0.0270 (13)	0.0280 (12)	−0.0001 (9)	0.0013 (9)	0.0066 (10)
C16	0.0213 (11)	0.0289 (12)	0.0227 (12)	0.0016 (10)	0.0017 (9)	0.0031 (10)
C17	0.0350 (14)	0.0257 (13)	0.0398 (15)	−0.0099 (11)	0.0013 (12)	−0.0001 (12)
O2	0.0227 (8)	0.0262 (9)	0.0218 (8)	−0.0026 (7)	0.0007 (7)	0.0017 (7)
N2	0.0184 (9)	0.0193 (10)	0.0202 (10)	−0.0010 (7)	−0.0036 (7)	−0.0004 (7)
C22	0.0226 (11)	0.0149 (11)	0.0277 (12)	−0.0015 (9)	−0.0062 (9)	−0.0008 (9)
C23	0.0198 (11)	0.0231 (12)	0.0278 (12)	0.0021 (9)	−0.0037 (9)	−0.0066 (10)
C24	0.0189 (11)	0.0292 (13)	0.0225 (11)	−0.0031 (10)	−0.0038 (9)	−0.0023 (10)
C25	0.0249 (11)	0.0177 (11)	0.0262 (12)	−0.0030 (9)	−0.0031 (10)	0.0024 (9)
C26	0.0214 (11)	0.0183 (11)	0.0246 (11)	0.0022 (9)	−0.0033 (9)	−0.0007 (9)
C27	0.0352 (14)	0.0400 (16)	0.0298 (14)	−0.0019 (13)	0.0035 (11)	0.0004 (12)

### Dichloridobis(4-methylpyridine N-oxide-κO)tin(II) (2) Geometric parameters (Å, º)

**Table:**

Sn1—O2	2.3078 (17)	C17—H17B	0.9800
Sn1—O1	2.4296 (17)	C17—H17C	0.9800
Sn1—Cl1	2.4939 (6)	O2—N2	1.340 (3)
Sn1—Cl2	2.5068 (6)	N2—C26	1.349 (3)
O1—N1	1.333 (3)	N2—C22	1.350 (3)
N1—C12	1.343 (3)	C22—C23	1.375 (4)
N1—C16	1.348 (3)	C22—H22	0.9500
C12—C13	1.377 (3)	C23—C24	1.393 (4)
C12—H12	0.9500	C23—H23	0.9500
C13—C14	1.390 (3)	C24—C25	1.393 (3)
C13—H13	0.9500	C24—C27	1.503 (4)
C14—C15	1.393 (3)	C25—C26	1.377 (4)
C14—C17	1.500 (3)	C25—H25	0.9500
C15—C16	1.376 (3)	C26—H26	0.9500
C15—H15	0.9500	C27—H27A	0.9800
C16—H16	0.9500	C27—H27B	0.9800
C17—H17A	0.9800	C27—H27C	0.9800
			
O2—Sn1—O1	169.66 (6)	C14—C17—H17C	109.5
O2—Sn1—Cl1	84.93 (4)	H17A—C17—H17C	109.5
O1—Sn1—Cl1	84.76 (4)	H17B—C17—H17C	109.5
O2—Sn1—Cl2	91.58 (5)	N2—O2—Sn1	122.98 (13)
O1—Sn1—Cl2	88.52 (5)	O2—N2—C26	119.6 (2)
Cl1—Sn1—Cl2	94.59 (2)	O2—N2—C22	119.37 (19)
N1—O1—Sn1	130.18 (14)	C26—N2—C22	121.0 (2)
O1—N1—C12	118.6 (2)	N2—C22—C23	120.0 (2)
O1—N1—C16	120.5 (2)	N2—C22—H22	120.0
C12—N1—C16	120.8 (2)	C23—C22—H22	120.0
N1—C12—C13	120.1 (2)	C22—C23—C24	121.0 (2)
N1—C12—H12	119.9	C22—C23—H23	119.5
C13—C12—H12	119.9	C24—C23—H23	119.5
C12—C13—C14	121.1 (2)	C25—C24—C23	117.1 (2)
C12—C13—H13	119.4	C25—C24—C27	121.4 (2)
C14—C13—H13	119.4	C23—C24—C27	121.5 (2)
C13—C14—C15	116.9 (2)	C26—C25—C24	120.7 (2)
C13—C14—C17	121.5 (2)	C26—C25—H25	119.6
C15—C14—C17	121.6 (2)	C24—C25—H25	119.6
C16—C15—C14	120.6 (2)	N2—C26—C25	120.2 (2)
C16—C15—H15	119.7	N2—C26—H26	119.9
C14—C15—H15	119.7	C25—C26—H26	119.9
N1—C16—C15	120.4 (2)	C24—C27—H27A	109.5
N1—C16—H16	119.8	C24—C27—H27B	109.5
C15—C16—H16	119.8	H27A—C27—H27B	109.5
C14—C17—H17A	109.5	C24—C27—H27C	109.5
C14—C17—H17B	109.5	H27A—C27—H27C	109.5
H17A—C17—H17B	109.5	H27B—C27—H27C	109.5
